# Supplementary material for: Association between Smoking and Urine Indole Levels Measured by a Commercialized Test
Source: Metabolites. 2022 Mar 9;12(3):234. doi: 10.3390/metabo12030234 (PMC8950635; doi:10.3390/metabo12030234)
Supplement: Supplementary file 1 [file metabolites-12-00234-s001.zip › metabolites-1581775-supplementary.pdf]

Supplemental Material

(No. of subjects)	Non-Smoking (94)	Past-Smoking (108)	Current-Smoking (71)	***P-Value***
Age (years)	45 ± 7	47 ± 4*	44 ± 4	***0.002***
BMI (kg/m^2)	24 ± 2	24 ± 2	24 ± 2	*0.942*
Circumference of Chest (cm)	94 ± 6	95 ± 5	94 ± 5	*0.499*
Circumference of Abdomen (cm)	82 ± 6	84 ± 6	83 ± 6	*0.21*
Systolic Blood Pressure (mmHg)	118 ± 13	115 ± 10	117 ± 12	*0.135*
Diastolic Blood Pressure (mmHg)	75 ± 9	73 ± 7	74 ± 10	*0.276*
WBC (×10^3/μL)	4.66 ± 0.97	4.69 ± 1.22	5.01 ± 1.18	*0.092*
RBC (×10^6/μL)	504 ± 33*	496 ± 35	486 ± 34	***0.004***
Hb (g/dL)	15.2 ± 0.83†	14.9 ± 0.81	15.0 ± 0.77	***0.033***
PLT (/μL)	22 ± 5	23 ± 5	23 ± 4	*0.724*
T-CHO (mg/dL)	199.6 ± 31.0	196.0 ± 30.0	191.1 ± 31.7	*0.202*
HDL-C (mg/dL)	60.7 ± 14.5	58.7 ± 13.7	60.5 ± 13.1	*0.161*
LDL-C (mg/dL)	118.1 ± 27.5	114.5 ± 26.6	109.6 ± 30.5	*0.531*
TG (mg/dL)	69.5(49.8, 92.5) †	79.5(60.3, 110.8)	72.0(54.0, 95.0)	***0.019***
AST (IU/L)	22.6 ± 5.3	25.4 ± 24.9	22.0 ± 5.3	*0.303*
ALT (IU/L)	21.7 ± 10.1	22.6 ± 13.2	20.8 ± 9.8	*0.571*
γ-GTP (IU/L)	33.1 ± 23.4	37.7 ± 29.1	36.1 ± 28.9	*0.478*
BUN (mg/dL)	15.0 ± 3.2	14.7 ± 3.4	14.0 ± 3.4	*0.113*
Cr (mg/dL)	0.98 ± 0.14	0.97 ± 0.11	0.97 ± 0.12	*0.814*
UA (mg/dL)	6.5 ± 1.3	6.4 ± 1.3	6.6 ± 1.4	*0.828*
FBS (mg/dL)	96.6 ± 7.6	98.9 ± 9.0	96.1 ± 9.6	*0.065*

IRI (µU/mL)	4.9 ± 2.0	5.1 ± 2.1	4.7 ± 1.9	*0.317*
HOMA-IR	1.19 ± 0.55	1.27 ± 0.54	1.12 ± 0.51	*0.227*
HOMA-beta	51.9 ± 17.4	53.3 ± 21.7	53.1 ± 23.5	*0.833*
HbA1C (%)	5.68 ± 0.25	5.75 ± 0.33	5.67 ± 0.30	*0.131*
CRP (mg/dL)	0.09(0.08, 0.12)	0.10(0.08, 0.12)	0.09(0.08, 0.13)	*0.484*
Previous history (Under treatment)				
Hypertension, n (%)	6 (6)	4 (4)	1 (1)	*0.267*
Hyperlipidemia, n (%)	5 (5)	5 (5)	0	*0.156*
Hyperuricemia, n (%)	12 (13)	9 (8)	3 (4)	*0.155*
Metabolic Syndrome, n (%)	2 (2)	6 (6)	4 (6)	*0.416*
NAFLD, n (%)	23 (25)	27 (25)	13 (18)	*0.539*
Alcohol intake (g/day)	0.91(0.40, 2.00) *	1.29(0.80, 2.37)	1.37(0.57, 2.74)	***0.045***

Table S1. Background characteristics of the subjects divided by smoking status
BMI, body mass index; WBC, white blood cells; RBC, red blood cells; Hb, hemoglobin; PLT, platelets; LDL, low density lipoprotein; TG, triglyceride; HDL, high density lipoprotein; AST, aspartate aminotransferase; ALT, alanine aminotransferase; γ-GTP, γ-glutamyltransferase; BUN, blood urea nitrogen; Cr, creatinine kinase; UA, uric acid; FBS, fasting blood sugar; IRI, immunoreactive insulin; HOMA, homeostasis model assessment; IR, insulin resistance; HbA1C, hemoglobin A1C; CRP, C-reactive protein; NAFLD, non-alcoholic fatty liver disease.

*; P<0.05 vs. current-smoker, †; P<0.05 vs. past-smoke.

Bold values show p < 0.05.

n = 273	β	P-Value
Circumference of Chest (cm)	0.033	0.584
Circumference of Abdomen (cm)	-0.009	0.879
Systolic Blood Pressure (mmHg)	0.071	0.243
Diastolic Blood Pressure (mmHg)	0.074	0.224
WBC ($\times 10^3/\mu L$)	0.000	0.999
RBC ($\times 10^6/\mu L$)	0.017	0.780
Hb (g/dL)	0.024	0.688
PLT (/μL)	-0.004	0.948
T-CHO (mg/dL)	-0.068	0.262
HDL-C (mg/dL)	0.017	0.776
LDL-C (mg/dL)	-0.044	0.466
AST (IU/L)	-0.093	0.125
FBS (mg/dL)	0.003	0.959
HOMA-IR	0.017	0.781
HOMA-beta	0.025	0.684
HbA1C (%)	-0.086	0.158
CRP (mg/dL)	-0.077	0.203
Previous history(Under treatment)		
Hyperuricemia, yes 1, no 0	0.014	0.816
Metabolic Syndrome, yes 1, no 0	0.021	0.734

Table S2. Univariate regression analysis for urine indole levels.
The factors are ones which were not included in Table 2.
Abbreviation as Table S1.
